# Supplementary material for: Microbial Composition of Extracted Dental Alveoli in Dogs with Advanced Periodontitis
Source: Microorganisms. 2024 Jul 17;12(7):1455. doi: 10.3390/microorganisms12071455 (PMC11278955; doi:10.3390/microorganisms12071455)
Supplement: Supplementary file 1 [file microorganisms-12-01455-s001.zip › Table S1.pdf]

Supplementary Table S1. Distribution of bacterial genera in samples from dog dental alveoli with periodontal disease

Taxonomy	%
d__Archaea;p_Methanobacteriota;o__Methanobacteriales;f_Methanobacteriaceae;g_Methanobrevibacter_A	1.9%
d__Bacteria;p__Actinobacteriota;o__Actinomycetales;f__Actinomycetaceae;g__Actinomyces	2.7%
d__Bacteria;p__Actinobacteriota;o__Actinomycetales;f__Actinomycetaceae;g__Buchananella	0.2%
d__Bacteria;p__Actinobacteriota;o__Actinomycetales;f__Actinomycetaceae;g__Pauljensenia	1.4%
d__Bacteria;p__Actinobacteriota;o__Coriobacteriales;f__Atopobiaceae;g__Olsenella	1.8%
d__Bacteria;p__Actinobacteriota;o__Coriobacteriales;f__Atopobiaceae;g__Olsenella_F	0.2%
d__Bacteria;p__Actinobacteriota;o__Coriobacteriales;f__Atopobiaceae;g__RUG721	1.2%
d__Bacteria;p__Actinobacteriota;o__Coriobacteriales;f__Eggerthellaceae;g__Slackia	0.0%
d__Bacteria;p__Actinobacteriota;o__Mycobacteriales;f__Mycobacteriaceae;g__Corynebacterium	2.5%
d__Bacteria;p__Actinobacteriota;o__Propionibacteriales;f__Propionibacteriaceae;g__Arachnia	0.8%
d__Bacteria;p__Bacteroidota;o__Bacteroidales;f__Bacteroidaceae;g__Alloprevotella	1.0%
d__Bacteria;p__Bacteroidota;o__Bacteroidales;f__Bacteroidaceae;g__Bacteroides	8.9%
d__Bacteria;p__Bacteroidota;o__Bacteroidales;f__Bacteroidaceae;g__Phocaeicola	0.1%
d__Bacteria;p__Bacteroidota;o__Bacteroidales;f__Bacteroidaceae;g__Prevotella	9.8%
d__Bacteria;p__Bacteroidota;o__Bacteroidales;f__Paludibacteraceae;g__F0058	0.1%
d__Bacteria;p__Bacteroidota;o__Bacteroidales;f__Paludibacteraceae;g__H1	0.1%
d__Bacteria;p__Bacteroidota;o__Bacteroidales;f__Porphyromonadaceae;g__Porphyromonas	31.2%
d__Bacteria;p__Bacteroidota;o__Bacteroidales;f__Porphyromonadaceae;g__Porphyromonas_A	4.3%
d__Bacteria;p__Bacteroidota;o__Bacteroidales;f__Tannerellaceae;g__Tannerella	5.6%
d__Bacteria;p__Bacteroidota;o__Flavobacteriales;f__Flavobacteriaceae;g__Capnocytophaga	0.3%
d__Bacteria;p__Bacteroidota;o__Flavobacteriales;f__Weeksellaceae;g__Bergeyella	0.2%
d__Bacteria;p__Campylobacterota;o__Campylobacterales;f__Campylobacteraceae;g__Campylobacter_A	1.2%
d__Bacteria;p__Chloroflexota;o__Anaerolineales;f__Anaerolineaceae;g__Flexilinea	1.0%
d__Bacteria;p__Desulfobacterota;o__Desulfobulbales;f__Desulfobulbaceae;g__Desulfobulbus	0.1%
d__Bacteria;p__Desulfobacterota_I;o__Desulfovibrionales;f__Desulfomicrobiaceae;g__Desulfomicrobium	0.8%
d__Bacteria;p__Desulfobacterota_I;o__Desulfovibrionales;f__Desulfovibrionaceae;g__Desulfovibrio	2.0%
d__Bacteria;p__Firmicutes;o__Erysipelotrichales;f__Coprobacillaceae;g__Eggerthia	0.2%
d__Bacteria;p__Firmicutes;o__Erysipelotrichales;f__Erysipelotrichaceae;g__Bulleidia	0.2%
d__Bacteria;p__Firmicutes;o__Erysipelotrichales;f__Erysipelotrichaceae;g__RQZE01	0.2%
d__Bacteria;p__Firmicutes;o__Lactobacillales;f__Streptococcaceae;g__Streptococcus	0.3%
d__Bacteria;p__Firmicutes_A;o__Lachnospirales;f__Vallitaleaceae;g__W11650	0.6%
d__Bacteria;p__Firmicutes_A;o__Oscillospirales;f__Oscillospiraceae;g__Evtepia	0.1%
d__Bacteria;p__Firmicutes_A;o__Peptostreptococcales;f__Anaerovoracaceae;g__Eubacterium_N	0.2%
d__Bacteria;p__Firmicutes_A;o__Peptostreptococcales;f__Anaerovoracaceae;g__Hornefia	1.0%
d__Bacteria;p__Firmicutes_A;o__Peptostreptococcales;f__Anaerovoracaceae;g__Mogibacterium	0.3%
d__Bacteria;p__Firmicutes_A;o__Peptostreptococcales;f__Filifactoraceae;g__Filifactor	1.4%
d__Bacteria;p__Firmicutes_A;o__Peptostreptococcales;f__Peptostreptococcaceae;g__Peptostreptococcus	4.3%

d__Bacteria;p__Firmicutes_A;o__Tissierellales;f__Peptoniphilaceae;g__Anaerosphaera	0.1%
d__Bacteria;p__Firmicutes_A;o__Tissierellales;f__Peptoniphilaceae;g__Parvimonas	0.4%
d__Bacteria;p__Firmicutes_A;o__Tissierellales;f__Peptoniphilaceae;g__W5053	0.9%
d__Bacteria;p__Firmicutes_C;o__Veillonellales;f__Dialisteraceae;g__Allisonella	0.0%
d__Bacteria;p__Firmicutes_C;o__Veillonellales;f__Dialisteraceae;g__Dialister	0.1%
d__Bacteria;p__Fusobacteriota;o__Fusobacteriales;f__Fusobacteriaceae;g__Fusobacterium	0.8%
d__Bacteria;p__Fusobacteriota;o__Fusobacteriales;f__Fusobacteriaceae;g__Fusobacterium_C	0.1%
d__Bacteria;p__Patescibacteria;o__Saccharimonadales;f__Saccharimonadaceae;g__Saccharimonas	0.0%
d__Bacteria;p__Proteobacteria;o__Burkholderiales;f__Burkholderiaceae;g__Lampropedia	1.2%
d__Bacteria;p__Proteobacteria;o__Burkholderiales;f__Burkholderiaceae;g__Ottowia	0.1%
d__Bacteria;p__Proteobacteria;o__Burkholderiales;f__Neisseriaceae;g__Eikenella	0.1%
d__Bacteria;p__Proteobacteria;o__Burkholderiales;f__Neisseriaceae;g__Neisseria	0.3%
d__Bacteria;p__Proteobacteria;o__Enterobacterales;f__Pasteurellaceae;g__Pasteurella	1.0%
d__Bacteria;p__Proteobacteria;o__Pseudomonadales;f__Moraxellaceae;g__Moraxella	0.0%
d__Bacteria;p__Proteobacteria;o__Xanthomonadales;f__Xanthomonadaceae;g__Lysobacter_B	0.2%
d__Bacteria;p__Spirochaetota;o__Treponematales;f__Treponemataceae;g__Treponema	0.8%
d__Bacteria;p__Spirochaetota;o__Treponematales;f__Treponemataceae;g__Treponema_B	0.6%
d__Bacteria;p__Spirochaetota;o__Treponematales;f__Treponemataceae;g__Treponema_C	0.1%
d__Bacteria;p__Spirochaetota;o__Treponematales;f__Treponemataceae;g__Treponema_D	0.1%
d__Bacteria;p__Synergistota;o__Synergistales;f__Aminobacteriaceae;g__CAJPSE01	4.9%
d__Bacteria;p__Synergistota;o__Synergistales;f__Aminobacteriaceae;g__Fretibacterium	0.2%
d__Bacteria;p__Synergistota;o__Synergistales;f__Dethiosulfovibrionaceae;g__Pyramidobacter	0.3%
