# Supplementary material for: Microbial Composition of Extracted Dental Alveoli in Dogs with Advanced Periodontitis
Source: Microorganisms. 2024 Jul 17;12(7):1455. doi: 10.3390/microorganisms12071455 (PMC11278955; doi:10.3390/microorganisms12071455)
Supplement: Supplementary file 1 [file microorganisms-12-01455-s001.zip › Table S2.pdf]

Supplementary Table S2. Distribution of bacterial species in samples from dog dental alveoli with periodontal disease

	Total abundance
Taxonomy	%
Methanobrevibacter_A oralis	1.8%
Methanobrevibacter_A smithii_A	0.0%
Actinomyces bowdenii	0.2%
Actinomyces bowdenii_B	0.3%
Actinomyces dentalis	0.5%
Actinomyces oricola	0.4%
Actinomyces sp001278845	0.8%
Actinomyces weissii	0.5%
Buchananella hordeovulneris	0.2%
Pauljensenia canis	0.7%
Pauljensenia cardiffensis	0.5%
Pauljensenia mediterranea	0.3%
Olsenella uli	1.8%
Olsenella_F sp001189515	0.2%
Atopobiaceae;g__RUG721;s__RUG721 sp004010535	1.2%
Slackia exigua	0.0%
Corynebacterium canis	2.1%
Corynebacterium freiburgense	0.3%
Corynebacterium mustelae	0.1%
Arachnia propionica_B	0.2%
Arachnia propionica_C	0.1%
Arachnia sp003932855	0.4%
Alloprevotella sp003859795	1.0%
Bacteroides heparinolyticus	2.2%
Bacteroides pyogenes	5.8%
Bacteroides pyogenes_A	0.9%

Phocaeicola abscessus	0.1%
Prevotella buccae	0.1%
Prevotella intermedia	0.4%
Prevotella koreensis	0.2%
Prevotella sp003932845	8.4%
Prevotella sp905372445	0.7%
Paludibacteraceae;g__F0058;s__F0058 sp000768855	0.1%
Paludibacteraceae;g__H1;s__H1 sp001653155	0.1%
Porphyromonas crevioricanis	4.4%
Porphyromonas gingivalis	1.0%
Porphyromonas gingivicanis	2.6%
Porphyromonas gulae	20.5%
Porphyromonas macacae	1.4%
Porphyromonas sp000768875	1.1%
Porphyromonas sp000769075	0.1%
Porphyromonas_A cangingivalis	2.6%
Porphyromonas_A canoris	1.6%
Tannerella forsythia	0.2%
Tannerella forsythia_A	5.3%
Capnocytophaga cynodegmi	0.3%
Bergeyella zoohelcum	0.2%
Campylobacter_A rectus	0.4%
Campylobacter_A sp004803855	0.0%
Campylobacter_A sp012978815	0.6%
Campylobacter_A sp013201975	0.2%
Flexilinea sp001717545	1.0%
Desulfobulbus oralis	0.1%
Desulfomicrobium orale	0.8%
Desulfovibrio sp003860215	2.0%
Eggerthia catenaformis	0.2%

Bulleidia moorei	0.2%
Erysipelotrichaceae;g__RQZE01;s__RQZE01 sp003858585	0.2%
Streptococcus anginosus	0.1%
Streptococcus constellatus	0.2%
Vallitaleaceae;g__W11650;s__W11650 sp002999035	0.1%
Vallitaleaceae;g__W11650;s__W11650 sp003858485	0.5%
Evtepia sp004556345	0.1%
Eubacterium_N saphenum	0.2%
Hornefia nodata	1.0%
Mogibacterium timidum	0.3%
Filifactor alocis	1.4%
Peptostreptococcus canis	4.3%
Anaerosphaera mikwangii	0.1%
Parvimonas micra	0.1%
Parvimonas sp000223315	0.3%
Peptoniphilaceae;g__W5053;s__W5053 sp000467935	0.9%
Allisonella pneumosintes	0.0%
Dialister invisus	0.1%
Fusobacterium animalis	0.1%
Fusobacterium canifelinum	0.3%
Fusobacterium nucleatum	0.1%
Fusobacterium nucleatum_J	0.2%
Fusobacterium sp000235465	0.2%
Fusobacterium_C necrophorum	0.1%
Saccharimonas sp013333795	0.0%
Lampropedia sp002285285	0.4%
Lampropedia sp003703475	0.8%
Ottowia sp003859965	0.1%
Eikenella shayeganii	0.1%
Neisseria animaloris	0.0%

Neisseria dumasiana	0.1%
Neisseria weaveri	0.1%
Neisseria zoodegmatis	0.0%
Pasteurella canis	0.1%
Pasteurella dagmatis	0.0%
Pasteurella multocida	0.3%
Pasteurella multocida_A	0.6%
Moraxella sp002224245	0.0%
Lysobacter_B sp002798195	0.2%
Lysobacter_B sp002798295	0.0%
Treponema medium	0.2%
Treponema sp000775995	0.0%
Treponema sp010365865	0.1%
Treponema sp018336815	0.1%
Treponema sp905372265	0.0%
Treponema sp905373565	0.3%
Treponema vincentii	0.0%
Treponema_B denticola	0.2%
Treponema_B denticola_A	0.3%
Treponema_B putidum	0.0%
Treponema_B sp905372345	0.1%
Treponema_C sp905372025	0.1%
Treponema_D socranskii	0.1%
Treponema_D sp014334325	0.0%
Aminobacteriaceae;g__CAJPSE01;s__CAJPSE01 sp003860125	4.6%
Aminobacteriaceae;g__CAJPSE01;s__CAJPSE01 sp905373185	0.2%
Fretibacterium fastidiosum	0.2%
Pyramidobacter piscolens	0.3%
